# Supplementary material for: Resolving the full spectrum of human genome variation using Linked-Reads
Source: Genome Res. 2019 Apr;29(4):635–45. doi: 10.1101/gr.234443.118 (PMC6442396; doi:10.1101/gr.234443.118)
Supplement: Supplemental Material [file supp_gr.234443.118_Supplemental_longranger-2.2.zip › longranger-2.2.tar.bz2!/lr2.2-public/lib/stan/cmdstan/stan/doc/api/html/check__range_8hpp_source.html]

Stan: src/stan/math/error\_handling/matrix/check\_range.hpp Source File


|  |  |
| --- | --- |
|  | Stan  2.5.0  probability, sampling & optimization |


- Main Page
- Related Pages
- Namespaces
- Classes
- Files

- File List
- File Members

All Classes Namespaces Files Functions Variables Typedefs Enumerations Enumerator Friends Macros Pages

- src
- stan
- math
- error\_handling
- matrix

check\_range.hpp

Go to the documentation of this file.

1 #ifndef STAN\_\_MATH\_\_MATRIX\_\_CHECK\_RANGE\_HPP

2 #define STAN\_\_MATH\_\_MATRIX\_\_CHECK\_RANGE\_HPP

3

4 #include <sstream>

5 #include <stdexcept>

6

7 namespace stan {

8  namespace math {

9

10  namespace {

11

12  void raise\_range\_error(size\_t max,

13  size\_t i,

14  const char\* msg,

15  size\_t idx) {

16  std::stringstream s;

17  s << "INDEX OPERATOR [] OUT OF BOUNDS"

18  << "; index=" << i

19  << "; lower bound=1"

20  << "; upper bound=" << max

21  << "; index position=" << idx

22  << "; " << msg

23  << std::endl;

24  throw std::out\_of\_range(s.str());

25  }

26

27  }

28

29  inline

30  void check\_range(size\_t max,

31  size\_t i,

32  const char\* msg,

33  size\_t idx) {

34  if (i < 1 || i > max)

35  raise\_range\_error(max,i,msg,idx);

36  }

37

38

39  }

40 }

41 #endif

stan::math::max

double max(const double a, const double b)

**Definition:** max.hpp:7

stan::math::check\_range

void check\_range(size\_t max, size\_t i, const char \*msg, size\_t idx)

**Definition:** check\_range.hpp:30


---

[ Stan Home Page ]

*© 2011–2014,
Stan Development Team.*
